# Entomopathogenic fungi as the microbial frontline against the alien *Eucalyptus* pest *Gonipterus platensis* in Brazil

**DOI:** 10.1038/s41598-021-86638-9

**Published:** 2021-03-31

**Authors:** Carolina Jordan, Paula Leite dos Santos, Leiliane Rodrigues dos Santos Oliveira, Mauricio Magalhães Domingues, Bianca Cristina Costa Gêa, Murilo Fonseca Ribeiro, Gabriel Moura Mascarin, Carlos Frederico Wilcken

**Affiliations:** 1 Departamento de Proteção Vegetal, Faculdade de Ciências Agronômicas, Universidade Estadual Paulista “Júlio de Mesquita Filho”, Botucatu, São Paulo 18610-307 Brazil; 2 Departamento de Medicina Interna, Faculdade de Medicina de Botucatu, Universidade Estadual Paulista “Júlio de Mesquita Filho”, Botucatu, São Paulo 18618-970 Brazil; 3 Empresa Brasileira de Pesquisa Agropecuária- Embrapa Meio Ambiente, Jaguariúna, São Paulo 13918-110 Brazil

**Keywords:** Fungi, Pathogens, Entomology

## Abstract

The eucalyptus snout beetle (ESB), *Gonipterus platensis*, is endemic to Australia but has become a major invasive, destructive pest of Brazilian eucalyptus plantations. Efforts to develop insecticides based on entomopathogenic fungi against ESB are limited by the lack of known virulent strains. We therefore explored the virulence of indigenous Brazilian strains of major entomopathogenic fungi—*Beauveria* spp. and *Metarhizium anisopliae*—against ESB adults. We found widely varying virulence and later capacities for conidial production on infected adult cadavers. Two strains stood out, *B. bassiana* IBCB-240 and *M. anisopliae* IBCB-364, as especially lethal for ESB adults under laboratory conditions, sporulated abundantly on infected insects, and also outperformed comparable strains used in commercial mycoinsecticides. Notably, *B. bassiana* IBCB-240 exhibited lower LT_50_ values at low inoculum levels (≤ 10^7^ conidia mL^−1^) and smaller LC_50_ values than *M. anisopliae* IBCB-364. Taken together, this study emphasizes natural variation in virulence among indigenous *Beauveria* and *Metarhizium* strains against ESB adults and identifies fungal strains with superior lethality to existing commercialized strains for managing this eucalyptus pest in Brazil.

## Introduction

In Brazil, *Eucalyptus* plantations provide wood production to many kinds of industries (pulp and paper, siderurgy, fiberboard panels, biomass, etc.), and are cultivated on 5.7 million hectares (ha) with an average productivity of 36 m^3^ ha^−1^ annually^1^. The *Eucalyptus* snout beetle (ESB), *Gonipterus platensis* Marelli (Coleoptera: Curculionidae), is an Australian defoliating pest present in Brazil since 1979 that, in 2003, caused damage to 50,000 ha of *E. grandis* x *E. urophylla* plantations^2,3^. Infestations of this pest can cause development of epicormic shoots, severe defoliation of the upper third of the tree crown and death of branches^4^. In Portugal, losses for wood productivity of *E. globulus* can reach 86%^5^, and the economic losses due to ESB in the last twenty years were estimated to be 648 million euros^6^.

Currently in Brazil, the forest plantations certified by such organizations as the Forest Stewardship Council (FSC) and National Forest Certification Program (Cerflor) occupy 3.5 million hectares^7^. These organizations recommend pesticide policies to reduce or eliminate uses of chemical pesticides and comply with local pesticide regulations. To date, no chemical insecticides have been registered in Brazil against this pest^8,9^. Therefore, biological control is the key management strategy adopted for *G. platensis* control, using natural enemies such as the egg parasitoid *Anaphes nitens* (Hymenoptera: Mymaridae)^10^, the predator *Podisus nigrispinus* (Hemiptera: Pentatomidae)^11^ and entomopathogenic nematodes^12^. In addition to these diverse biocontrol agents, entomopathogenic fungi are the largest commercially utilized microbial biocontrol agents in Brazil, but few effective fungal strains have been studied for use against ESB^9^. Based on their safety for humans and other non-target organisms^13^, and their low risk to elicit insect resistance^14^, entomopathogenic fungi are uniquely able to invade the host insects directly through the integument and thus can be used as an additional component in the integrated management toolbox against ESB in eucalyptus plantations. Indeed, these mycoinsecticides are environmentally friendly alternatives or even complementary to chemical insecticides where millions of hectares in Brazil are treated annually with *B. bassiana* and *M. anisoliae* for controlling forest and agricultural arthropod pests^9^.

Commercial preparations of *B. bassiana* and *M. anisopliae* were first used to control *Gonipterus* sp. n. 2 in South Africa under laboratory conditions, with *Beauveria* showing higher efficiency by contact and ingestion^15^; *B. bassiana* was later reported to induce epizootics in *G. platensis* in Itararé, SP, Brazil^16^. This fungus is compatible with *A. nitens* releases due to its low infectivity for this parasitoid^17^. Field applications of mycoinsecticides require a set of environmental conditions suitable for inoculation and colonization in the host, so during the dry season the control efficiency drops to less than 20%^18^. To date, in Brazil, there are only two commercial mycoinsecticides based on *B. bassiana* officially available for ESB control: Boveril (ESALQ-PL63 strain) and Ballvéria (IBCB-66 strain)^8^. However, these commercial fungal strains may not be suitable or virulent enough to control ESB effectively and are not so used.

This research screened indigenous Brazilian strains of *M. anisopliae* and *Beauveria* spp. for improved activity against ESB adults under laboratory conditions as a first and crucial step in developing more effective mycoinsecticides, and we compared the most promising strains to those in the commercial mycoinsecticides registered for ESB control in Brazil. This is the first comprehensive fungal screening for the biological control potential against this invasive pest, and raises possibilities for much enhanced control of ESB in Brazilian eucalyptus plantations.

## Results

### Fungal strain selection against *G. platensis*

**Figure 1:**
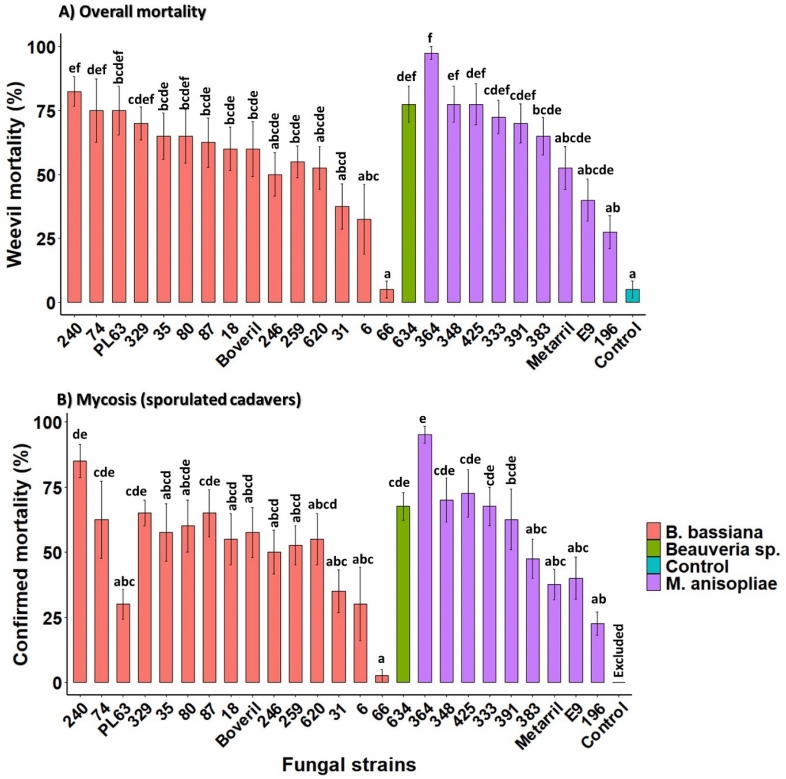
Cumulative mortality rate by day 20 post-spraying (**A**) and confirmed mortality (i.e., sporulated cadavers or mycosis rate [**B**]) of *G. platensis* adults after inoculation with 1 × 10^8^ conidia mL^−1^ of several strains of *M. anisopliae*, *Beauveria* sp. and *B. bassiana*. Untreated weevils from control group were sprayed only with Tween-80 solution (0.1%). Vertical bars represent means (± SE, n = 8 replicates with a total of 40 weevils per treatment), while different letters denote significant contrasts between treatments by Tukey HSD method (*P* < 0.05). There are four commercial strains explored in Brazil and used here as positive controls for the sake of comparison: *B*. *bassiana* IBCB-66 (only unformulated) and ESALQ-PL63 (unformulated and formulated as Boveril); *M. anisopliae* ICBC425 (only unformulated) and ESALQ-E9 (unformulated and formulated as Metarril).

The screening revealed wide variation of pathogenicity and virulence among fungal strains and also demonstrated a remarkable phenotypic plasticity of *G. platensis* (ESB) adults regarding fungal infection (*χ*^2^ = 126.04, df = 25, *P* < 0.0001). The average mortality rates due to *B. bassiana* strains and another unidentified *Beauveria* strain ranged between 5 and 85%, while *M. anisopliae* strains inflicted mortalities in the range of 27.5–95% (Fig. 1A). According to the overall mortality analysis, the most pathogenic strains were *B. bassiana* IBCB-240 and *M. anisopliae* IBCB-364, inducing 85% and 95% deaths in ESB adults at 20 days post-spraying, respectively. Commercial mycoinsecticides based on Boveril and Metarril killed 60% and 52.1% of ESB adults, while their unformulated strains, ESALQ-PL63 and ESALQ-E9, promoted mortality rates of 65.9% and 32.5%, respectively. In this sense, *M. anisopliae* IBCB-364 outperformed Metarril and its unformulated strain, ESALQ-E9, whereas *B. bassiana* IBCB-240 did not statistically differ from Boveril or its strain ESALQ-PL63 (Fig. 1A). The lowest mortality rate, as low as 5%, was achieved with *B. bassiana* IBCB-66, the active ingredient of the commercial product Ballvéria.

The proportion of infected, sporulating cadavers remarkably varied among fungal strains (*χ*^2^ = 115.92, df = 24, *P* < 0.0001). The mycosis rate induced by *B. bassiana* and *Beauveria* sp. ranged from 2.5 to 85%, while *M. anisopliae* caused mycosis in the range of 22.5 to 95% (Fig. 1B). The greatest numbers of sporulating cadavers were observed with *M. anisopliae* IBCB-364 (95% sporulated insects) and *B. bassiana* IBCB-240 caused 85% mycosis. The sporulation rate was lowest with *B. bassiana* IBCB-66 (2.5%) and *M. anisopliae* IBCB-196 (22.5%), whereas the remaining strains exhibited moderate levels of mycosis. Control mortality averaged 12.5 ± 8.4% and showed no evidence of fungal infection. A high percent mortality did not always coincide with a high *post-mortem* sporulation rate. In this sense, while 75% of weevils were killed by *B. bassiana* ESALQ-PL63, only 30% of those cadavers showed external sporulation, thus indicating that the fungus might not complete its life cycle in the host.

**Figure 2:**
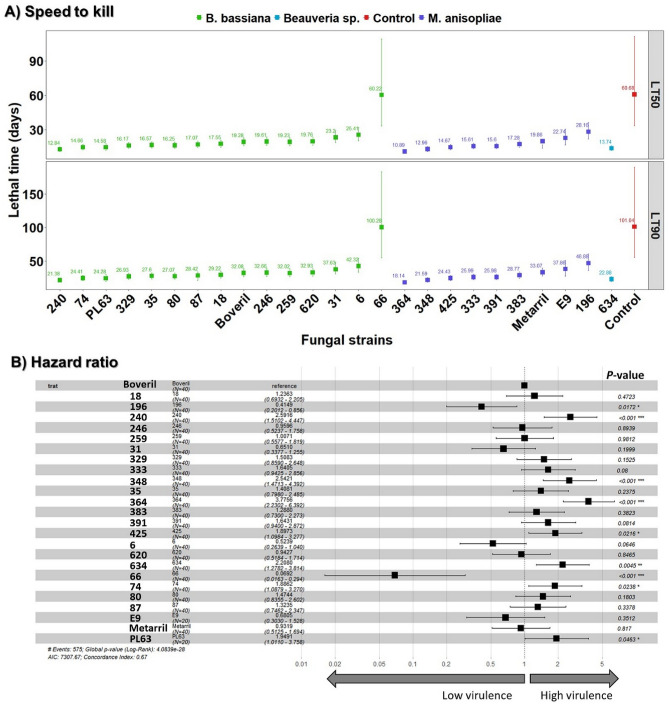
Susceptibility of *G. platensis* adults after exposure to several strains of *M. anisopliae* and *B. bassiana* applied with a single concentration of 1 × 10^8^ conidia mL^−1^. (**A**) Speed to kill represented by 50% and 90% lethal times (LT_50_/LT_90_, in days) estimated with Weibull models fitted to survival censored data of several fungal strains. Lethal times are significantly different when their 95% confidence intervals (whiskers) do not overlap. (**B**) Hazard ratios (square symbols) for fungal infection compared to the commercial mycoinsecticide Boveril, which is assigned as the reference group and currently registered to control *Gonipterus* spp. in Brazil. Significant *P*-values are indicated by asterisks (*< 0.05, **< 0.01 or ***< 0.001) and represent the strains that were significantly different (more/less virulent) from the reference Boveril. Whiskers represent the 95% CIs.

All strains of *Beauveria* spp. and *M. anisopliae* killed ESB adults, although the rate at which this happened differed significantly (Supplemental Fig. S1). The survival analysis revealed the susceptibility of ESB adults to different fungal strains tested at a single concentration used as a discriminatory baseline (*χ*^2^ = 250.59, df = 25, *P* < 0.0001). All pairwise comparisons between ESB survival curves after exposure to fungal strains are described in Supplemental Table S1. Cox regression analysis based on hazard ratios (HR) comparing controls (untreated ESB) with all test strains underscored that most *M. anisopliae* and *Beauveria* strains significantly increased the risk of insects dying (*χ*^2^ = 246.4, df = 25, *P* < 0.0001) (Supplemental Fig. S2). Accordingly, weevils exposed to highly virulent strains exhibited mortality risks of 55.6 and 38.12 times greater than insects from control group when exposed to *M. anisopliae* IBCB-364 and *B. bassiana* IBCB-240. Conversely, the only exception was noted for *B. bassiana* IBCB-66 which portrayed a similar hazard ratio and survival pattern to control (Supplemental Table S1 and Fig. S1-S2; log-rank *P* = 0.988). *B. bassiana* IBCB-6 and *M. anisopliae* IBCB-196 mortality rates were less than 50%, thereby categorized as low virulence candidates (Supplemental Fig. S1, S2). The two hihgly virulent strains (IBCB-240 and IBCB-364) significantly reduced ESB lifespan resulting in LT_50_’s of 12.8 and 10.9 days and LT_90_’s of 21.4 and 18.1 days post-treatment, respectively, with a concentration of 1 × 10^8^ conidia mL^−1^ (Fig. 2A). In agreement with mortality and mycosis best results, insects exposed to both *B. bassiana* IBCB-240 and *M. anisopliae* IBCB-364 showed similarly the sharpest decrease in survival rates among all fungi tested (log-rank *P* = 0.214, Supplemental Table S1, Fig. S1). The remaining strains exhibited low or moderate virulence profiles (Supplemental Fig. S1, S2). On the other hand, ESB adults from control groups and those exposed to the less virulent strains also survived longer with estimated LT_50_ and LT_90_ values exceeding 25 and 40 days, respectively (Fig. 2A).

Hazard ratios (HR) were estimated relative to the mortality pattern of the reference strain (ESALQ-PL63) using its commercial formulation Boveril. Using the classification proposed by Valero-Jiménez et al.^19^, 17 strains were classified as lethal, four strains fell into the highly lethal (HL) category, and three were classified as slowly lethal (SL) strains (Fig. 2B). Virulence varied widely: strains *M. anisopliae* IBCB-364 and *B. bassiana* IBCB-240 were on average 3.8 and 2.6 times, respectively, more virulent, while *B. bassiana* IBCB-66 was on average 14 times (i.e., 1/0.069) less virulent than the reference Boveril.

### Conidial production by sporulated cadavers

**Figure 3:**
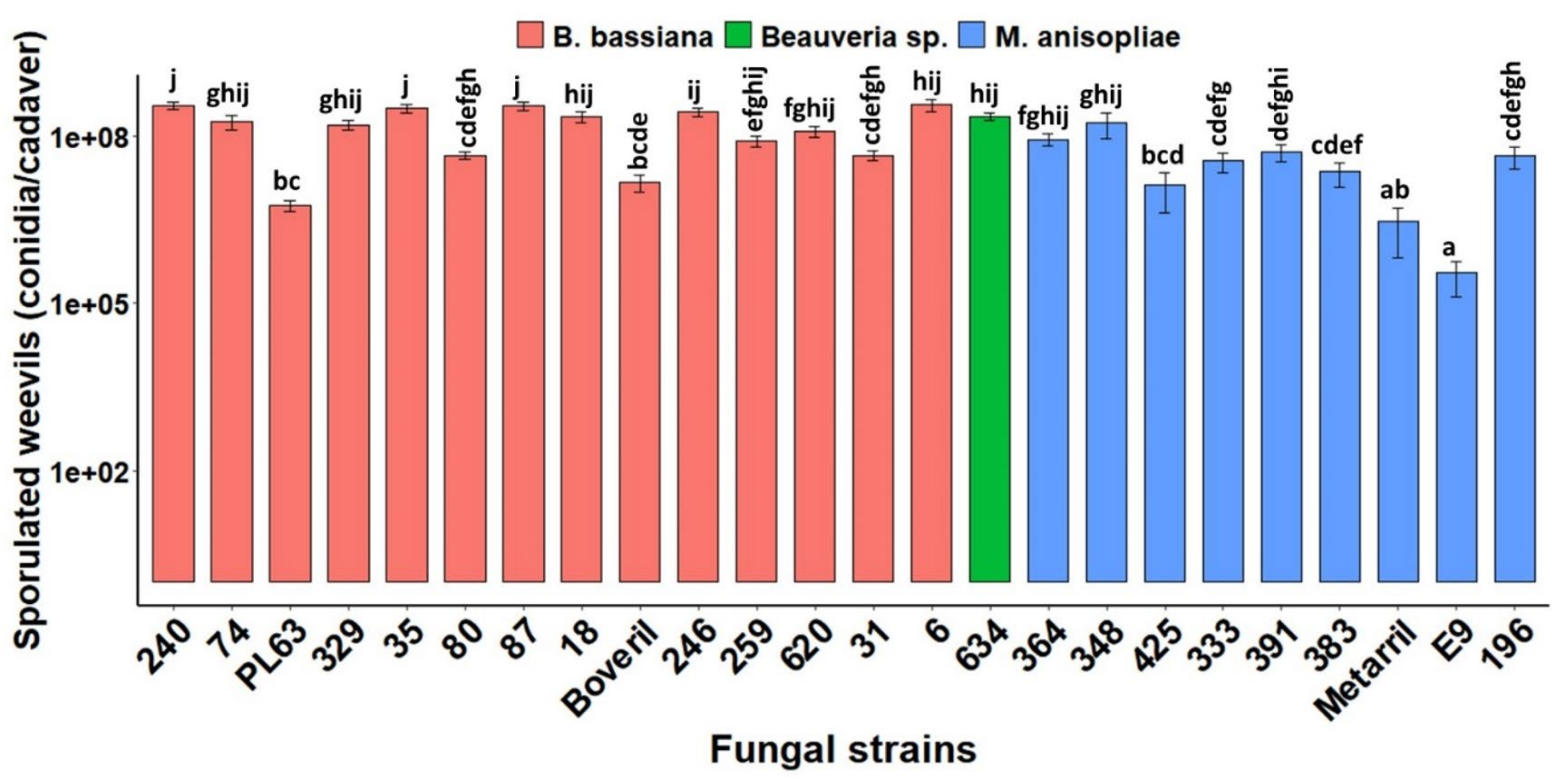
Secondary inoculum production by infected weevils’ cadavers of various strains of *M. anisopliae* and *B. bassiana* after incubation in saturated humid chambers. Data were fitted to a generalized linear model with negative binomial distribution, and estimated means (± SE, n = 10 replicates) were statistically separated by Tukey HSD test at *P* < 0.05. Note that *B. bassiana* IBCB-66 was not included in this analysis, as it had one weevil that sporulated.

Conidial production on infected cadavers varied strongly among fungal strains (*χ*^2^ = 308.40, df = 23, *P* < 0.0001), with *B. bassiana* and *Beauveria* sp. strains producing the largest amounts of conidia per cadaver. The lowest post-mortem conidial production was achieved with *M. anisopliae* ESALQ-E9 (2.5 × 10^5^ conidia weevil^−1^), whilst the highest yields were attained by *B. bassiana* strains IBCB-18, IBCB-240, IBCB-246, IBCB-329, IBCB-6, IBCB-35, IBCB-620, IBCB-87 and *Beauveria* sp. IBCB-634, which all produced more than 10^8^ conidia per cadaver (Fig. 3). For *M. anisopliae* strains, IBCB-348 and IBCB-364 gave the highest conidial production on cadavers with a range of 5.8–9.3 × 10^7^ conidia per adult. Moreover, *B. bassiana* IBCB-66 had only one individual that sporulated (2.8 × 10^7^ conidia adult^−1^) post-mortem but also showed poor virulence, and was not considered further in this analysis. Conidial production on cadavers by *B. bassiana* IBCB-240 and *M. anisopliae* IBCB-364, the two most virulent strains, did not differ statistically. Sporulated (mycosed) insects infected by either *M. anisopliae* or *B. bassiana* showed especially typical fungal outgrowth through the intersegmental membranes followed by a profuse conidiogenesis (Supplemental Fig. S3).

Strains depicting the highest virulence do not necessarily yielded higher conidial production on infected cadavers. The higher overall mortality rates of the most virulent strains, IBCB-364 and IBCB-240, also showed higher mycelial growth/sporulation (mycosis) rates (Correlation coefficient *r* = 0.89, *P* < 0.0001) and lower LT_50_ and LT_90_ values (*r* = -0.98, *P* < 0.0001), yet no significant relationship was found with inoculum production in infected beetles (*r* = 0.36, *P* = 0.19), according to the Spearman’s correlation test (Supplemental Fig. S4).

### Virulence of the two strains most lethal to *G. platensis*

**Figure 4:**
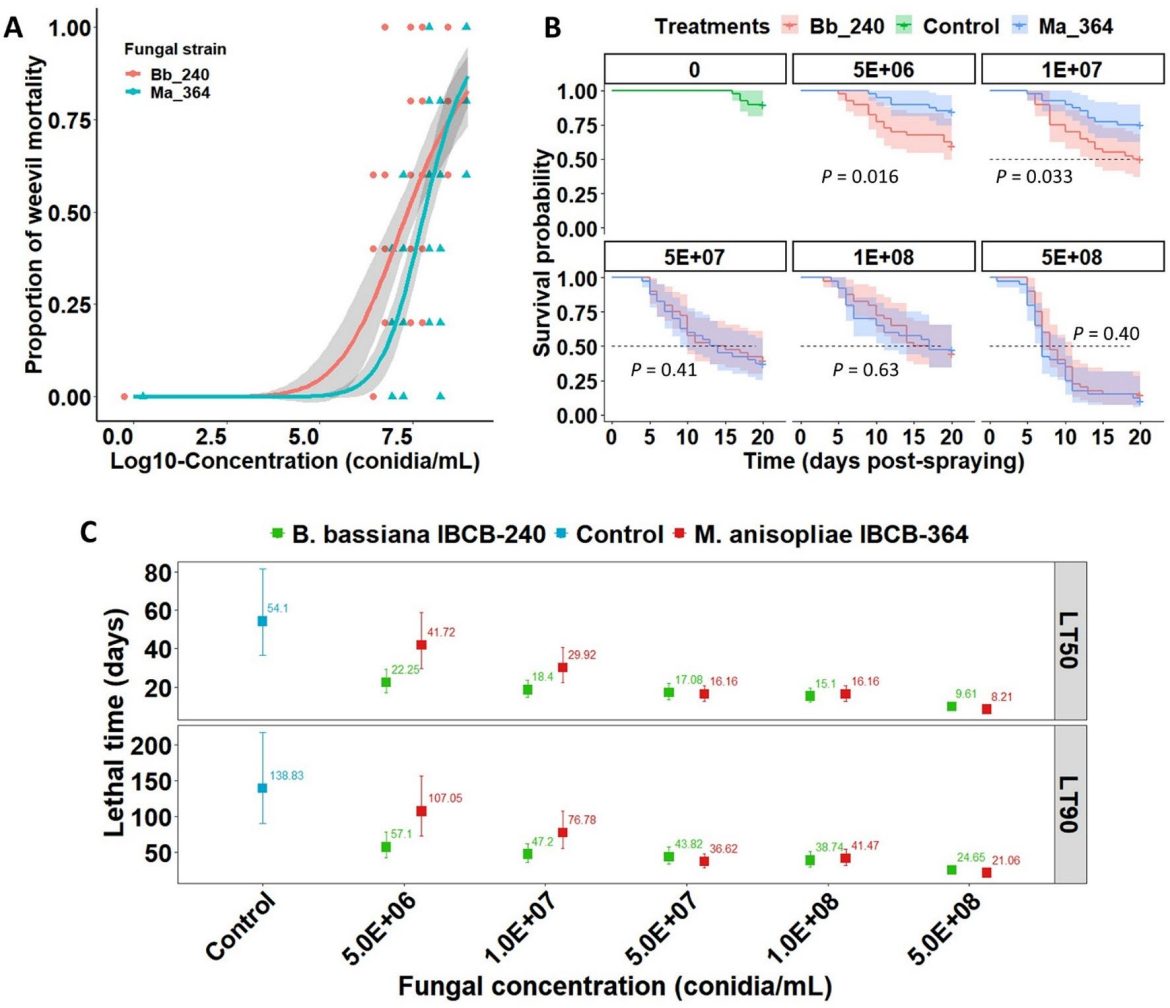
Comparative virulence between strains IBCB-240 (*B. bassiana*) and IBCB-364 (*M. anisopliae*) against *G. platensis* adults assessed with time-concentration-mortality bioassays. (**A**) Concentration-mortality response curves at 15 days post-spraying fitted with a two-parameter log-logistic models (solid lines are fitted models, grey bands are 95% confidence intervals and points with different shapes are observational data). (**B**) Kaplan–Meier survival curves of *G. platensis* adults exposed to two fungal strains and compared with log-rank test (*P* < 0.05) within each conidial concentration tested. Log-rank *P*-values for all the pairwise comparisons between survival curves are presented in Table S2. (**C**) Estimated median (LT_50_) and 90% (LT_90_) lethal times with their corresponding 95% confidence intervals (whiskers) for *G. platensis* adults after being sprayed with five conidial concentrations of each fungal strain. Statistical differences between LT_50_ or LT_90_ values are indicated when their 95% confidence intervals do not overlap.

Virulence of the highly lethal (HL) strains of *B. bassiana* and *M. anisopliae* showed a strong concentration–time-effect against adult *G. platensis*, in which mortality rate increased with increasing concentration and time (Supplemental Fig. S5). The values for LC_50_ at 15 days post-infection and LT_50_/LT_90_ obtained for different spore concentrations were determined. When examining the concentration-mortality data on 15 days post-spraying, *B. bassiana* IBCB-240 was more virulent than *M. anisopliae* IBCB-364, especially at the lower applied concentrations, as also supported by the significant difference between their concentration-mortality curves (*χ*^2^ = 12.17, df = 2, *P* = 0.0023) (Fig. 4A). In addition, the slopes of the mortality curves of *B. bassiana* IBCB-240 and *M. anisopliae* IBCB-364 differed significantly (*t* = 1.997, *P* = 0.046), suggesting that *G. platensis* adults displayed different susceptibilities to both fungal strains (Table 1). In agreement with these results, *B. bassiana* IBCB-240 required a threefold reduction in conidial concentration to kill 50% of weevils in relation to *M. anisopliae* IBCB-364, indicating that the former is indeed more virulent at lower inoculum level (*t* = 2.26, *P* = 0.026) (Table 1). With respect to mycosis (sporulation) rates, infected adults showed 100% confirmed mortality with either strain.

**Table 1:** Two-parameter log-logistic model fitted to concentration-mortality data for describing the virulence of two fungal strains to *G. platensis* adults in terms of their estimated median lethal concentrations (LC_50_) with corresponding 95% confidence intervals. Concentration is expressed as conidia mL^-1^.

Fungal strain	n*	Model parameters		*t* (*P*-value)^§^	Median lethal concentrations (conidia mL^−1^) ^¥^
		Inflection point (*e*) ± SE	Slope (*b*) ± SE^†^		LC_50_ (95% ci)
*B. bassiana* ICBC-240	240	7.37 ± 0.16	− 7.79 ± 1.73 a	4.50 (< 0.0001)	2.33 (1.12–4.86) × 10^7^ a
*M. anisopliae* ICBC-364	240	7.82 ± 0.13	− 13.35 ± 2.18 b	6.13 (< 0.0001)	6.62 (3.75–11.69) × 10^7^b

*Total number of insects in the experiment.

^†^Slope for mortality represents regression of proportion of weevils versus log (concentration) of conidia. Different letters indicate significant contrast between slopes according to Student t-test (*P* < 0.05).

^**§**^Student t-test and *P*-values represent the probability of slope ≠ 0.

^**¥**^Student t-test contrasts for estimated lethal concentrations indicate significant differences when followed by distinct letters at *P* < 0.05.

According to the survival analysis, the lifespan of *G. platensis* decreased as conidial concentrations increased (*χ*^2^ = 72.73, df = 1, *P* < 0.0001) and was strongly reduced in the presence of these strains (*χ*^2^ = 19.34, df = 2, *P* < 0.0001). After 20 days of incubation, the control weevils exhibited a mortality rate of 12.5 ± 6.5% without any signs of fungal mycelial growth and sporulation. The Kaplan–Meier survival analysis revealed virulence differences between strains of *B. bassiana* and *M. anisopliae* according to their survival curves when tested at lower inoculum levels (≤ 10^7^ conidia mL^-1^) (Fig. 4B). In addition, LT_50_/LT_90_ values considerably decreased with increasing conidial concentration (Fig. 4C). ESB adults exposed to lower concentrations of *M. anisopliae* IBCB-364 (5 × 10^6^ and 10^7^ conidia mL^−1^) had survivorships similar to untreated control insects (log-rank *P* = 0.50 and *P* = 0.08, respectively, Supplemental Table S2), indicating that this strain was not virulent for this weevil at those inoculum levels (Fig. 4B). This finding is further shown by longer lethal times induced by *M. anisopliae* and the control group with no differences in their LT_50_/LT_90_ values (Fig. 4C). Conversely, at these same low dosages, survival curves differed between fungal strains, indicating *G. platensis* lifespan after exposure to *B. bassiana* IBCB-240 decreased faster than when exposed to *M. anisopliae* IBCB-364 (log-rank *P* = 0.016 and *P* = 0.033, respectively) (Fig. 4B, Table S2). This also means that LT_50_/LT_90_ values differed significantly between both strains, with lower values attained by *B. bassiana* IBCB-240 (Fig. 4C). Nonetheless, similar adult survival curves were achieved with higher concentrations, 5 × 10^7^ to 5 × 10^8^ conidia mL^−1^, for both fungi (log-rank *P* = 0.41, *P* = 0.63 and *P* = 0.40, respectively) (Fig. 4B, Supplemental Table S2), in which LT_50_ values fell to 8.2–29.9 days while LT_90_ values ranged from 21 to 36.6 days (Fig. 4C).

## Discussion

Eleven of 15 *Beauveria* strains and one of eight *M. anisopliae* strains tested here (including the highly lethal strains IBCB-240 and IBCB-364) originated from curculionid host species, suggesting a pre-adaptation of these fungi to *G. platensis*. Overall, 21 of 23 tested fungal strains significantly reduced weevil survival starting at day 5, with *B. bassiana* IBCB-240 and *M. anisopliae* IBCB-364 exhibiting the lowest LT_50_/LT_90_ values, between 11 and 21 days while inducing the highest mortality and mycosis levels in a faster rate than the other fungal strains tested in this study. Among all these strains, only four of them fell in the highly lethal (HL) category when compared to the reference mycoinsecticide Boveril, but our primary focus emphasized the two top strains that outperformed the others in all evaluated parameters, including in vivo conidial production as a important factor for horizontal transmission within the context of disease development. Other fitness parameters that may be affected by sub-lethal doses were not investigated in the current study, although further research should address them within the context of fungus-weevil interaction^20,21^.

The cuticle is the primary physical and chemical barrier that fungal entomopathogens must overcome to infect the host^22^. Because *B. bassiana* IBCB-66 performance did not differ significantly from the untreated control group, we assumed this fungus either encountered difficulties penetrating the weevil or that infections were blocked by the host’s immune system. Some antimicrobial cuticular secretions and the cuticular thickness of *G. platensis* might confer some natural resistance to penetration and/or infection by some strains of generalist fungal entomopathogens, as indicated here by the low virulence of *B. bassiana* IBCB-66, which may require higher fungal exposures to infect and to kill weevils. This observation agrees with the study of Ramirez et al.^23^ that some *Cordyceps* (formerly *Isaria*) spp. strains with minimal pathogenicity to mosquitoes could not complete cuticular penetration or infection of these hosts. Such cuticular resistance is strongly related to detrimental effects to conidia of *B. bassiana* and *M. anisopliae* by cuticular lipids and aldehydes^24,25^. From a broader perspective, our results about the widely-varying virulence for ESB by a diversity of fungal entomopathogens may reflect a co-evolutionary host/pathogen ‘arms race’ and provide a useful model system to study the multifactorial complexities of interactions between a fungus and its host on the cuticle surface; in addition, if penetration succeeds, there is another battle between the pathogen and the host’s immune system by allowing or blocking a lethal infection. The low virulence and low mortality rates of some *B. bassiana* and *M. anisopliae* strains against *G. platensis* suggest that ESB may be able to resist many generalist fungal pathogens.

However, due to host phenotypic plasticity and natural genetic variation among strains of a single fungal species^19,26^, host origin cannot guarantee strain virulence; *Beauveria* and *Metarhizium* possess famously broad host spectra and innumerable ecological adaptations^27^. This was especially true for the least pathogenic strains, including *B. bassiana* IBCB-66, IBCB-6 and IBCB-31, which all came from weevil hosts but produced slow infection and poor mortality rates in *G. platensis* (Figs. 1, 2).

The wide genetic plasticity and broad lifestyle options of these two fungi coupled with their large genomes (size ~ 33–39 Mb) provide diversity among their strains as well as highly variable degrees of virulence to different hosts, which represents a valuable resource of biocontrol candidates for use in pest control and comprising the majority of registered commercial mycoinsecticides worldwide^27,28^. One plausible explanation for the results obtained with the time-dose-mortality response bioassays between *M. anisopliae* and *B. bassiana* can be drawn from comparing their genomes for species-specific virulence genes and gene family expansions and contractions that might correlate with host ranges and complex multifaceted pathogenic strategies. It has been shown that *B. bassiana* contains many more gene products similar to or derived from bacterial toxins than most other fungi that may give some ecological advantages over other entomopathogenic fungal species to access a wide variety of hosts^29^. Accordingly, highly expressed virulence genes unique to *B. bassiana* strain (e.g., bacterial-like toxins) might provide some comparative advantage over *M. anisopliae* against *G. platensis,* even at low inoculum dosages, as it was observed in this study.

Post-mortem production of conidia has not been investigated previously in *G. platensis* and plays an important role for fungal secondary infection through horizontal transmission, recycling in the host’s environment, and disease establishment in host population. In our study, we demonstrated that strains of *Beauveria* spp. and *M. anisopliae* differed greatly in their capability to sporulate on host cadavers, and that this emergent sporulation from cadavers confirmed the successful infection and completion of the fungal life cycle in this host, with the majority of fungal strains being able to produce conidia on cadavers. Notably, the highest sporulation rates occurred in the highly lethal (HL) group of strains (Fig. 1). Exceptionally, only one individual infected by the low-virulence strain *B. bassiana* IBCB-66 exhibited profuse sporulation, which seems that even after significant lethal infections the fungus was unable to complete the sporulation phase in the host for unknown reasons. Despite the remarkably variable capacity for in vivo conidial production among fungal strains, remarkably more conidia were produced on cadavers infected by *Beauveria* spp. than by *M. anisopliae*. On most infected *G. platensis* cadavers, subsequent fungal growth and sporulation was limited to intersegmental membranes, mainly those involving the mouthparts, antennae, compound eyes, legs, and under the elytra (Supplemental Fig. S3). It has been postulated that a fungus produces large numbers of infective propagules could enhance fungal population density in host environments and the likelihood of further dispersal in the host population^30^. Comparing both highly lethal strains in this study, *B. bassiana* IBCB-240 strain produced 4 times more conidia per cadaver than *M. anisopliae* IBCB-364; this, in turn, may indicate that this *B. bassiana* strain is better adapted to weevil’s natural habitat and because of its copious sporulation on cadavers can provide enough propagules for effective microbial control and dispersal to enable secondary infections in the host population. In agreement with our results, it was observed that *M. anisopliae* strains induced lower total sporulation on termite cadavers than *B. bassiana* strains, but the former exhibited abundant rapid sporulation, which emphasizes distinct sporulation patterns between these fungal species on different insect hosts and also underscores their distinct ecological adaptations^31^.

Our results revealed the highly virulent strains to be up to 3.8 times more effective and to produce higher in vivo sporulation than the strain used in the commercial Boveril bioinsecticide registered for ESB control in Brazil (Figs. 1, 2, 3). The dose-dependent effects of spore concentration have already been established^32^. It is especially important that the highly lethal strain *B. bassiana* IBCB-240 yielded a stronger dose–effect and more rapid reduction of survival rates for *G. platensis* than *M. anisopliae* IBCB-364 when tested with lower concentrations (≤ 10^7^ conidia mL^−1^) (Fig. 4, Table 1). In this sense, reducing the required effective dosage would lessen the costs of production and application while also shortening exposures to non-target hosts.

Beyond the virulence traits addressed in the present study, additional efforts should be placed on determining the most tolerant fungal strains to heat, dehydration (osmotic stress), UV-tolerance, mass production of spores, as well as bioefficacy under field applications before developing a commercial mycoinsecticide against this insect pest. Taken all together, the present study paves the way for the development of an effective mycoinsecticide against *G. platensis* in Brazil that has minimal negative impact on the environment and beneficial insects^33^. After examining a significant range of fungal strains, the highly virulent strains chosen here, *B. bassiana* (IBCB-240) and *M. anisopliae* (IBCB-364), appear to be excellent candidates for the development of new mycoinsecticides for use in a commercial biological control program specifically designed to manage this invasive pest in Brazilian eucalyptus plantations. Prospectively, additional research addressing the compatibility of these two virulent strains with the egg-parasitoid wasp *A. nitens* deserves special attention to establish a truly integrated strategy to deploy both biocontrol agents for ESB control in eucalyptus plantations.

## Conclusion

In summary, these results constitute a major step in developing environmentally safe biocontrol entomopathogenic fungi to manage this invasive weevil pest. In addition, the two most lethal strains selected here could be used together with a novel *Gonipterus*-associated microsporidium pathogen recently identified by our group that naturally infects ESB populations in Brazil, or safely applied after the release of egg-parasitoid wasp (*A. nitens*) in field populations; both strategies could potentially contribute to mitigate the burden of this pest as well as to minimize any likelihood of host resistance to a high-virulence strain. Finally, the widely variable natural virulence demonstrated among strains of *M. anisopliae* and *B. bassiana* offers the opportunity to better understand the molecular and genetic mechanisms underlying this heterogeneity and to support the potential development of improved biocontrol strains with desired properties.

## Methods

### Source and rearing of *G. platensis*

Insects were maintained at the Laboratory for Biological Control of Forest Pests (LCBPF), School of Agricultural Sciences, (FCA), São Paulo State University (UNESP), Campus of Botucatu, under controlled conditions (25 ± 1 °C, RH: 50 ± 10% and 12 h photoperiod). The colony was begun with *G. platensis* adults collected in outbreaks in São Paulo and Paraná states. The species identification was confirmed by taxonomic characters^34^.

These adults were fed on fresh shoots of *Eucalyptus urophylla*, obtained from the same site where this study took place (UNESP, Botucatu, SP, Brazil), with their stems immersed in plastic containers containing water, changed once a week. Insect colonies were kept in rearing cages (40 cm long × 45 cm wide × 80 cm high), with glass roof and sides of voile fabric.

### Source, culture, viability and deposition rate of fungi

We assayed 13 strains of *B. bassiana,* one strain of *Beauveria* sp., seven strains of *M. anisopliae*, and the active strains from two commercial products, Boveril WP (Koppert Ltda., Piracicaba, SP, Brazil) based on *B. bassiana* ESALQ-PL63 (used here as reference strain), which is registered to control *Gonipterus* spp.; and also the commercial product Metarril WP (Koppert Ltda., Piracicaba, SP, Brazil) based on *M. anisopliae* strain ESALQ-E9. All other fungal strains were originally obtained from the Collection of Entomopathogenic Microorganisms “Odemar Cardim Abreu” at the Experimental Center of the Instituto Biológico, Campinas, SP, or otherwise re-isolated and purified from commercial fungal formulations (Table 2) were preserved at − 20 °C in 10% glycerol. Additional seed-cultures to serve as inoculum for bioassays were kept in test tubes containing Potato Dextrose Agar (PDA, 42 g L^−1^ [Sigma-Aldrich, St. Louis, MO, USA]) and stored at 6 °C. Such seed cultures of each strain were grown in Petri dishes (90 × 15 mm) containing PDA (15 mL per plate) in a climatic growth chamber at 25 ± 1 °C with 12 h photoperiod for 14 days until complete development and sporulation (conidiogenesis).

**Table 2:** Origin, date of collection, host and species of fungal strains native to Brazil used in this study. All fungal strains used in the current study are registered at the National System for the Management of Genetic Heritage and Associated Traditional Knowledge—SisGen under the code AA51E70.

Species	Strain	Host	Date of collection	Geographical origin	Geographical coordinates
*Beauveria bassiana*	IBCB-6	*Cosmopolites sordidus*	February 01, 1986	Miracatu—SP	24°17′13.2″S 47°27′28.1″W
	IBCB-18	*Hypothenemus hampei*	November 01, 1987	Tapiratiba—SP	21°29′03.8″S 46°46′10.2″W
	IBCB-31	*Nezara viridula*	March 01, 1986	Piracicaba—SP	22°43′25.0″S 47°38′35.5″W
	IBCB-35	*Cosmopolites sordidus*	March 01, 1986	Cruz das Almas—BA	12°39′06.1″S 39°07′17.8″W
	IBCB-66	*Hypothenemus hampei*	September 01, 1986	São José do Rio Pardo—SP	21°35′46.0″S 46°53′17.5″W
	IBCB-74	*Aphidoidea*	March 01, 1988	Campinas—SP	22°54′10.8″S 47°03′52.9″W
	IBCB-80	*Hypothenemus hampei*	December 01, 1988	Caconde—SP	21°31′14.5″S 46°38′40.2″W
	IBCB-87	*Cosmopolites sordidus*	April 01, 1989	Goiânia—GO	16°40′08.4″S 49°16′00.5″W
	IBCB-240	*Hypothenemus hampei*	June 01, 1999	Campinas—SP	22°54′05.0″S 47°04′05.2″W
	IBCB-246	*Hypothenemus hampei*	June 01, 1999	Taubaté—SP	23°01′40.4″S 45°33′22.0″W
	IBCB-259	*Hypothenemus hampei*	June 01, 1999	Taubaté—SP	23°01′40.4″S 45°33′22.0″W
	IBCB-329	Native forest	June 01, 1999	Guaraniaçu—PR	25°05′34.8″S 52°52′18.1″W
	IBCB 620	*Hypothenemus hampei*	August 08, 2008	São Paulo—SP	23°32′24.0″S 46°38′22.9″W
	ESALQ-PL63	*Atta* sp.	January 01, 1992	Piracicaba—SP	22°42′45.27″S 47°37′38.5″W
*Beauveria* sp.	IBCB-634	*Epacroplon cruciatum*	May 23, 2009	Cel . Macedo—SP	23°38′15.7″S 49°18′49.0″W
*Metarhizium anisopliae*	IBCB-196	Soil	May 01, 1999	Piracicaba—SP	22°43′56.6″S 47°38′56.4″W
	IBCB-333	*Mahanarva fimbriolata*	June 01, 1999	Valparaíso—SP	21°13′42.2″S 50°52′01.9″W
	IBCB-348	*Mahanarva fimbriolata*	June 02, 1999	Sertãozinho—SP	21°08′38.4″S 48°00′41.8″W
	IBCB-364	*Anthonomus grandis*	July 14, 1999	Araras—SP	22°21′32.0″S 47°22′47.6″W
	IBCB-383	*Mahanarva fimbriolata*	July 01, 1999	Araras—SP	22°21′32.0″S 47°22′47.6″W
	IBCB-391	*Mahanarva fimbriolata*	August 02, 1999	Tabapuã—SP	20°57′27.0″S 49°01′37.6″W
	IBCB-425	Soil	January 05, 2000	Iporanga—SP	24°35′11.4″S 48°35′41.3″W
	ESALQ-E9	*Mahanarva posticata*	May 05, 1981	Boca da Mata—MT	9°40′1.73″S 36°07′23.04″W

*All fungal strains were deposited in the microbial germplasm named Collection of Entomopathogenic Microorganisms “Odemar Cardim Abreu” at the Experimental Center of the Instituto Biológico, Campinas, SP, Brazil, with a prefix code ‘IBCB’. Fungal strains ESALQ-PL63 and ESALQ-E9 are proprietary of Koppert do Brasil Holding Ltd. (Piracicaba, SP, Brazil) and were re-isolated and purified in PDA from serial dilutions of their respective commercial formulated products named Boveril WP and Metarril WP, respectively.

The collection, maintenance, and use in bioassays of these fungal isolates and the studied insect specimen were conducted in accordance with the Brazilian National System of Management of Genetic Heritage and Associated Traditional Knowledge—referred to as “SisGen” is approved under the protocol # AA51E70.

The viability of all test fungi was determined by spreading a 100-μL aliquot of a conidial suspension (10^6^ conidia mL^−1^), prepared with sterile surfactant solution (0.1% v/v) of Tween 80, on PDA medium (PDA 42 g L^−1^) in Petri dishes (90 × 15 mm) and incubated in the dark at 25 ± 1 °C. Plates of *Beauveria* and *Metarhizium* were incubated for 16 and 18 h, respectively, prior to evaluation. Conidia were scored as viable if any germ tube was 2× longer than the diameter of the spore; a total of 100 conidia per sample were scored under 400× magnification in a phase-contrast microscope (Leica Microsystems, DM 500, Heerbrugg, Switzerland)^35^. Fungal strains presenting ≥ 90% viability were used in the insect bioassays.

**Figure 5:**
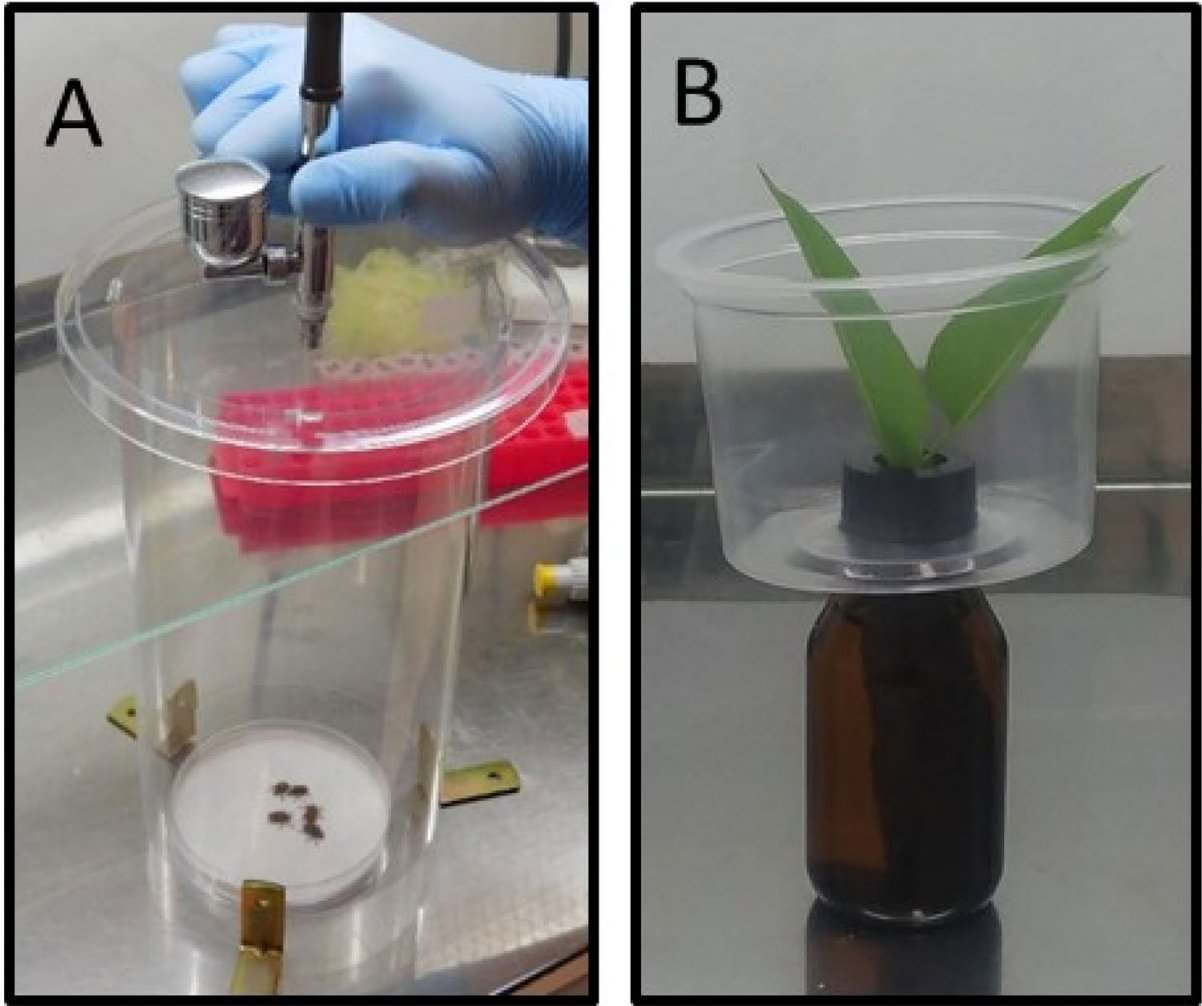
Detail of the experiment set up for screening fungal strains against adults of *G. platensis* under laboratory conditions. (**A**) Portable mini-sprayer assembled with a dual-gravity airbrush, modified from Mascarin et al. (2013), operated with 10 PSI and loaded with 127 µL of fungal suspension for application onto *G. platensis* beetles. Acrylic cylinder measured 11.5 cm in diameter and 23 cm in height. The triplet created a free space of 2 cm to allow depressurization during spraying. (**B**) Experimental unit consisting of eucalyptus leaf with its petiole immersed in a glass vial filled with water to keep its turgor and serving to feed the beetles confined in a plastic container.

The inoculum deposition rate was assessed by a portable microspray tower. The concentration of 10^8^ conidia mL^−1^ tested in the screening study was prepared in sterile Tween-80 solution (0.1%). The aqueous suspension was then sprayed onto 7 glass coverslips (20 × 20 mm) evenly placed on a plastic Petri dish (90 × 15 mm). The volume of spore suspension was chosen in accordance with the field application rate of 200 L ha^-1^ and sprayed on a 63.6 cm^2^ Petri dish area. Then an aliquot of 127 µL of conidial suspension of each fungal strain was loaded in the reservoir of a professional dual-gravity airbrush DB134K (Fenghua Bida Machinery Manufacture Co., China), and applied to the coverslips from about 25 cm away from its nozzle and using 10 PSI working pressure (adapted from Mascarin et al.^36^) (Fig. 5A). The experiment was carried out in duplicate on different occasions, with three repetitions per treatment. After spraying, all 7 coverslips were transferred to 15-mL centrifuge tubes containing 5 mL of 0.1% Tween-80. This suspension was vortexed vigorously for 2 min to forcefully dislodge conidia from coverslips. Each suspension was quantified with a hemocytometer (Neubauer chamber, HGB, Precicolor, Germany) at 400 × magnification.

### Fungal screening bioassay (single concentration)

We assayed 14 strains of *B. bassiana,* one strain of *Beauveria* sp. and eight strains of *M. anisopliae* towards *G. platensis* adults. Conidia were produced in plastic Petri dishes containing PDA (42 g L^−1^) at 25 ± 1 °C in total darkness. Bioassays were done with conidia harvested from 14-d-old cultures on PDA by superficial scraping with a Drigalski spatula; germination (viability) was assessed as noted above to be ≥ 90% prior to each bioassay.

Conidial suspensions were prepared with sterile Tween-80 solution (0.1% v/v) at 10^8^ conidia mL^−1^. The control group consisted of ESB adults sprayed with a sterile solution of Tween-80 (0.1%). In parallel, commercially-produced fungal strains as pure cultures and as their formulated products were used as standard treatments.

For each fungal strain, a conidial suspension of 127 µL containing 10^8^ conidia mL^−1^ (equivalent to 143.0–287.5 conidia mm^−2^ within 18–21 s) was sprayed onto insects in four independent repetitions using the portable micro-sprayer, inside a biological safety cabinet. Each biological repetition consisted of 5 ESB adults in a petri dish (90 × 15 mm) lined with a sterile filter paper at the bottom. Each replicate was independently sprayed with each fungal suspension, and all fungal strains were assayed at the same time in each experiment.

After spraying fungal treatments, the insects remained for 24 h in Petri dishes in a growth chamber at 25 ± 1 °C, 83.0 ± 2.0% RH with 12 h photophase to ensure the residual contact of the fungus with insects. This methodology afforded two routes of contamination of this insect by fungi: (1) direct contamination by topical spore deposition on the cuticle, and (2) indirect contamination through tarsal contact on a surface treated with the fungal conidia (Fig. 5B).

At 24 h after spraying, adults of *G. platensis* were transferred to plastic pots and fed with leaves of *E. urophylla* (clone 433) whose petioles were placed in hydrated phenolic foam to maintain leaf turgor. Leaves were replaced as needed. The incubation conditions were the same as described above. Adults were monitored on a daily basis during 20 days for assessing dead and living individuals. Dead insects were removed and superficially disinfected with sodium hypochlorite solution (1% v/v) for 30 s and subsequently rinsed twice in sterile distilled water for 30 s. Afterwards, dead insects were dried at room temperature and then individually transferred to wells of moist chambers made with 24-well culture plates. These cadavers were incubated at the same conditions above for more 15 days and assessed for fungus sporulation (i.e., emerging hyphae and spores) to verify mortalities caused by the applied fungus.

The experiment consisted of 26 treatments all 23 fungal strains, with 1 control, and two commercial formulated products as standards. Each treatment consisted of 4 independent repetitions, with 5 adults each, totalling 20 insects (sex ratio 1:1) per treatment, and the entire experiment was repeated twice on different occasions (blocks) using new fungal batches and insect cohorts to ensure data reproducibility. Virulence of fungal strains was evaluated according to the percentage of overall mortality as well as the median and 90% lethal times (LT_50_ and LT_90_) for *G. platensis* obtained from survival analysis after exposure to each fungal strain.

### Quantification of conidial production in vivo

After incubating dead individuals in humid chamber for 14 days, five adults covered with conidia (sporulated cadavers) were randomly removed from each sample of fungal treatment, surface sterilized with sodium hypochlorite (1% v/v) for 30 s, rinsed twice in sterile distilled water for 30 s, and then cadavers were placed in a humid chamber maintained at 26 °C with 14 h photophase for 14 days. Cadavers bearing conspicuous masses of aerial conidia were placed individually in 1.5-mL microcentrifuge (Eppendorf) tubes filled with 1 mL of sterile solution of 0.1% Tween-80 and vigorously vortexed for 1 min to dislodge conidia from insect’s body. Subsequently, these same tubes were vortexed vigorously for 1 min and left in an ultrasonic bath for 2 min to enhance the suspension of conidia. Conidial density was calculated by haemocytometer counts at 40 × magnification to determine the average of conidial production per insect. Two repeated assays were used for each fungal strain, totalling 10 cadavers per treatment. Altogether, the conidial production was measured in 230 cadavers (23 treatments × 2 experimental replicates × 5 cadavers).

### Concentration–response assays with best strains

*M. anisopliae* IBCB-364 and *B. bassiana* IBCB-240 were previously selected for further evaluations of their virulence based on in terms of LC_50_ and LT_50_ and LT_90_ obtained from concentration–time-mortality bioassays using multiple conidial concentrations. Fungal suspensions containing conidia retrieved from PDA cultures were prepared and applied as described above. Experimental units consisted of 5 adults per plastic pot (repetition), with 4 repetitions per treatment (i.e., n = 20 adults with sex ratio 1:1). The strains were tested at concentrations of 5 × 10^6^, 10^7^, 5 × 10^7^, 10^8^, and 5 × 10^8^ conidia mL^-1^. After spraying fungal treatments likewise mentioned, beetles were kept in controlled conditions (25 ± 1 °C, 83.0 ± 2.0% RH and 12 h photophase). Each repetition was monitored daily for up to 20 days; dead insects were recorded, removed and transferred to moist chambers to induce outgrowth and sporulation as a means to confirm mortality. Viability tests were performed on PDA as described previously*,* with both strains attaining viability > 90%. The experiment included a control group (as described above). Each treatment and 4 repetitions involved 20 insects. All fungal concentrations were assayed at once in each experiment, and repeated twice at different times (blocks) using new fungal batches and insect cohorts. ESB adults were fed with fresh leaves of *E. urophylla* (clone 433) and replaced as needed. The virulence of these two strains were compared according to their concentration-mortality curves and respective estimated lethal concentrations (LC_50_), and their estimated survival curves and respective estimated lethal times (LT_50_ and LT_90_) for each fungal concentration.

### Data analysis

All experimental designs followed a completely randomized arrangement, and experiments were always repeated at least twice on different dates to ensure data reproducibility. Cumulative mortality data for *G. platensis* for up to 20 days after application with fungal entomopathogens, as well as percent confirmed mortality data were separately fitted to a generalized linear model (GLM) with binomial distribution for errors and logit link function, in which fixed effects were attributed to fungal strains and experimental blocks in the linear predictor. The only exception is that values for mycosis in control group (no fungus) was “zero” for all replicates, and was dropped from the analysis. A total of 8 biological replications from two repeated experiments (blocks) were performed for each treatment (fungal strain). Once the treatment effect was significant, multiple pairwise comparisons of means were carried out using Tukey HSD method at 5% significance (*P* < 0.05) to separate fungal strains, using the package “emmeans”^37^. Regarding the mortalities recorded over time as well as for each conidial concentration tested in both screening and virulence bioassays, censored survival probability data on *G. platensis* adults were fitted to parametric Weibull and log-normal models, respectively, using the *survreg* function that enabled the estimation of median (50%) and 90% lethal times (LT_50_ and LT_90_). In addition, survival curves constructed with the non-parametric Kaplan–Meier method were further pairwise comparisons were made between fungal strains within each conidial concentration based on the log-rank test at *P* < 0.05, using the function *pairwise_survdiff*. Differences between the control and the infected weevils were examined using a Cox proportional hazards model based on the risk of weevil death and respective hazard ratios (HR; the daily chance of death), implemented with the *coxph* function and visualized them using the *ggforest*. In addition, hazard ratios (HR) of insects exposed to different fungal strains in comparison with the commercial product Boveril were calculated. The survival analysis was performed with the “survival”^38^ and “survminer”^39^ packages.

Secondary inoculum production by sporulated cadavers was fitted to a generalized linear mixed model with Poisson distribution and log link function to account for overdispersion, using the package “lme4”^40^. Fixed effects were assigned to fungal strains and experimental blocks, including random effects for the observational level in the linear predictor. Means were compared according to Tukey HSD test at *P* < 0.05, using the package “emmeans”^37^. As a complimentary analysis, the cluster analysis was performed to overall mortality, mycosis, LT_50_/LT_90_ estimates and in vivo inoculum production by infected insects to facilitate visualization of similar and dissimilar fungal strains tested in the screening study. Predicted means derived from model fits were normalized prior to turning into distances based on the Euclidean method, a measure of similarity. Subsequently, Euclidean distances were subjected to a hierarchical clustering tree (in rows and columns) taking into account all variables and fungal strains using Ward’s method. The heat map was computed and described using a function of heatmap.2 in the “gplots” package^41^. To determine the type of relationship between these response variables, average data on overall mortality rate was correlated with mycosis rate, LT_50_, LT_90_ and in vivo inoculum production using Spearman’s correlation method that allowed to compute *r* coefficient and *P*-value.

Proportion data for a specific time interval after spraying from multiple concentration-mortality response bioassays involving two fungal strains were fitted with a two-parameter log-logistic model with binomial distribution using the package “drc”^42^. The following model fitted to the mortality-concentration data can be written as:

$$y= c+\frac{d-c}{1+exp(b\left(\mathrm{log}\left(x\right)-\mathrm{log}\left(e\right)\right))}$$

where *y* is the mortality probability, *b* is the slope (steepness of the curve), *e* is the inflection point, while lower limit *c* is zero (i.e., natural mortality in control was null) and upper limit *d* is equal to 1. We chose to analyse data on 15-day mortality for *G. platensis* exposed to these fungi, because most fungal concentrations had reached maximum mortality levels while control group had no natural mortality. Estimated effective median concentration (LC_50_) for each fungal strain were extracted from these dose–response models along with their corresponding robust standard errors (using function *sandwich*) and the delta method for 95% confidence intervals adjusted for over-dispersion (i.e., multiplication by a scaling factor). Model slopes (*b*, curve steepness) and estimated LC_50_ values were compared using Student t-test to identify significant contrasts at *P* < 0.05, while difference between dose–response curves was assessed with the log-likelihood ratio test.

All graphs were plotted with package “ggplot2”^43^. Goodness-of-fit of models was assessed using half-normal plots with simulation envelopes^44^ or Akaike’s Information Criterion (AIC) along with residual plot examination. All analyses were performed in the statistical environment R^45^.

## Supplementary Information

Supplementary Information

## Data Availability

The datasets generated during and/or analyzed during the current study are available from the corresponding author on reasonable request.
