# Supplementary material for: Entomopathogenic fungi as the microbial frontline against the alien Eucalyptus pest Gonipterus platensis in Brazil
Source: Sci Rep. 2021 Mar 31;11:7233. doi: 10.1038/s41598-021-86638-9 (PMC8012584; doi:10.1038/s41598-021-86638-9)
Supplement: Supplementary file 1 — Supplementary Information [file 41598_2021_86638_MOESM1_ESM.docx]

**Supplementary Material**

**Entomopathogenic fungi as the microbial frontline against the alien *Eucalyptus* pest *Gonipterus platensis* in Brazil**

Carolina Jordan^a*^, Paula Leite dos Santos^a^, Leiliane Rodrigues Dos Santos Oliveira^c^, Mauricio Domingues Magalhães^a^, Bianca Cristina Costa Gêa^a^, Murilo Fonseca Ribeiro, Gabriel Moura Mascarin^b*^, Carlos Frederico Wilcken^a^

^a^Departamento de Proteção Vegetal, Faculdade de Ciências Agronômicas, Universidade Estadual Paulista “Júlio de Mesquita Filho”, 18610-307, Botucatu, São Paulo, Brazil

^b^Departamento de Medicina Interna, Faculdade de Medicina de Botucatu, Universidade Estadual Paulista “Júlio de Mesquita Filho”, 18618-970, Botucatu, São Paulo, Brazil

^c^Empresa Brasileira de Pesquisa Agropecuária- Embrapa Meio Ambiente, 13918-110, Jaguariúna, São Paulo, Brazil

*Correspondence to: **Gabriel M. Mascarin**, e-mail: [gabriel.mascarin@embrapa](mailto:gabriel.mascarin@embrapa); [gmmascar@gmail.com](mailto:gmmascar@gmail.com). **Carolina Jordan**, e-mail: carolina.jordan@unesp.br.

**Table S1.** *P*-values adjusted with Bonferroni-Holmes were obtained from pairwise comparisons of survival curves of *G. plantesis* adults exposed to different fungal strains using log-rank test at *P* < 0.05 (significant *P*-values are highlighted in bold). Data refer to screening bioassays using single concentration of 1 × 10^8^ conidia mL^-1^.

|  | **18** | **196** | **240** | **246** | **259** | **31** | **329** | **333** | **348** | **35** | **364** | **383** | **391** | **425** | **6** | **620** | **634** | **66** | **74** | **80** | **87** | **Boveril** | **Control** | **E9** | **Metarril** |
| --- | --- | --- | --- | --- | --- | --- | --- | --- | --- | --- | --- | --- | --- | --- | --- | --- | --- | --- | --- | --- | --- | --- | --- | --- | --- |
| **196** | **0.00507** | - | - | - | - | - | - | - | - | - | - | - | - | - | - | - | - | - | - | - | - | - | - | - | - |
| **240** | **0.01799** | 3.3e-07 | - | - | - | - | - | - | - | - | - | - | - | - | - | - | - | - | - | - | - | - | - | - | - |
| **246** | 0.47166 | **0.05079** | **0.00244** | - | - | - | - | - | - | - | - | - | - | - | - | - | - | - | - | - | - | - | - | - | - |
| **259** | 0.57402 | **0.02334** | **0.00198** | 0.92298 | - | - | - | - | - | - | - | - | - | - | - | - | - | - | - | - | - | - | - | - | - |
| **31** | 0.10738 | 0.36433 | **8.8e-05** | 0.38733 | 0.28388 | - | - | - | - | - | - | - | - | - | - | - | - | - | - | - | - | - | - | - | - |
| **329** | 0.55034 | **0.00055** | 0.08586 | 0.19998 | 0.22003 | **0.02455** | - | - | - | - | - | - | - | - | - | - | - | - | - | - | - | - | - | - | - |
| **333** | 0.40659 | **9.4e-05** | 0.14162 | 0.11573 | 0.12146 | **0.00907** | 0.81990 | - | - | - | - | - | - | - | - | - | - | - | - | - | - | - | - | - | - |
| **348** | **0.02200** | **3.5e-06** | 0.96753 | **0.00501** | **0.00501** | **0.00032** | 0.11747 | 0.21625 | - | - | - | - | - | - | - | - | - | - | - | - | - | - | - | - | - |
| **35** | 0.72256 | **0.00117** | **0.03220** | 0.30451 | 0.31989 | **0.04521** | 0.80644 | 0.65376 | **0.03875** | - | - | - | - | - | - | - | - | - | - | - | - | - | - | - | - |
| **364** | **9.1e-05** | **5.6e-11** | 0.21419 | **6.5e-06** | **2.2e-06** | **9.0e-08** | **0.00122** | **0.00271** | 0.23085 | **0.00032** | - | - | - | - | - | - | - | - | - | - | - | - | - | - | - |
| **383** | 0.94784 | **0.00169** | **0.02219** | 0.39331 | 0.45047 | 0.05988 | 0.64794 | 0.47436 | **0.03781** | 0.75843 | **7.9e-05** | - | - | - | - | - | - | - | - | - | - | - | - | - | - |
| **391** | 0.42950 | **0.00018** | 0.16525 | 0.12601 | 0.13045 | **0.01272** | 0.81351 | 0.99529 | 0.23009 | 0.63318 | **0.00501** | 0.49335 | - | - | - | - | - | - | - | - | - | - | - | - | - |
| **425** | 0.16884 | **9.8e-06** | 0.33132 | **0.03921** | **0.03786** | **0.00211** | 0.46899 | 0.66283 | 0.43643 | 0.35163 | **0.01401** | 0.21843 | 0.67004 | - | - | - | - | - | - | - | - | - | - | - | - |
| **6** | **0.02219** | 0.63562 | **3.9e-06** | 0.15432 | 0.10239 | 0.65441 | **0.00364** | **0.00135** | **1.6e-05** | **0.00740** | **1.7e-09** | 0.01318 | **0.00181** | **0.00020** | - | - | - | - | - | - | - | - | - | - | - |
| **620** | 0.44873 | **0.03701** | **0.00092** | 0.94628 | 0.87822 | 0.38204 | 0.15436 | 0.08139 | **0.00252** | 0.22003 | **8.7e-07** | 0.35163 | 0.08742 | **0.02409** | 0.15479 | - | - | - | - | - | - | - | - | - | - |
| **634** | 0.07024 | **4.9e-06** | 0.68224 | **0.01401** | **0.01409** | **0.00074** | 0.23624 | 0.36108 | 0.72256 | 0.12916 | 0.08139 | 0.08742 | 0.36909 | 0.63562 | **4.6e-05** | **0.00696** | - | - | - | - | - | - | - | - | - |
| **66** | **1.4e-06** | **0.01997** | **2.7e-11** | **4.5e-05** | **9.8e-06** | **0.00163** | **6.3e-08** | **6.5e-09** | **2.6e-10** | **2.0e-07** | **1.3e-15** | **2.3e-07** | **1.9e-08** | **5.4e-10** | **0.00612** | **2.2e-05** | **3.1e-10** | - | - | - | - | - | - | - | - |
| **74** | 0.19998 | **2.2e-05** | 0.33206 | **0.04247** | **0.04789** | **0.00288** | 0.50223 | 0.66283 | 0.35684 | 0.34815 | **0.01434** | 0.21691 | 0.66917 | 0.96212 | **0.00022** | **0.02822** | 0.62915 | **1.7e-09** | - | - | - | - | - | - | - |
| **80** | 0.62915 | **0.00106** | 0.06287 | 0.22003 | 0.25670 | **0.03450** | 0.94429 | 0.77789 | 0.07024 | 0.87260 | **0.00104** | 0.65376 | 0.77111 | 0.43674 | **0.00501** | 0.17556 | 0.19998 | **1.8e-07** | 0.45047 | - | - | - | - | - | - |
| **87** | 0.85631 | **0.00293** | **0.02219** | 0.39319 | 0.46353 | 0.07851 | 0.65441 | 0.47815 | **0.02782** | 0.89264 | **0.00011** | 0.94311 | 0.49135 | 0.21742 | **0.01397** | 0.36108 | 0.08139 | **4.1e-07** | 0.26330 | 0.79561 | - | - | - | - | - |
| **Boveril** | 0.53966 | **0.02367** | **0.00177** | 0.94216 | 0.96292 | 0.29689 | 0.20492 | 0.11608 | **0.00335** | 0.29595 | **1.6e-06** | 0.44483 | 0.12916 | **0.03322** | 0.10184 | 0.92972 | **0.01143** | **9.8e-06** | **0.04026** | 0.24148 | 0.44382 | - | - | - | - |
| **Control** | **7.9e-07** | **0.01702** | **1.5e-11** | **3.0e-05** | **5.9e-06** | **0.00135** | **3.3e-08** | **3.3e-09** | **2.0e-10** | **1.0e-07** | **4.2e-16** | **1.2e-07** | **9.3e-09** | **2.6e-10** | **0.00501** | **1.3e-05** | **2.0e-10** | 0.98771 | **8.0e-10** | **1.0e-07** | **2.5e-07** | **6.0e-06** | - | - | - |
| **E9** | 0.20366 | 0.36130 | **0.00239** | 0.50545 | 0.43674 | 0.95867 | 0.08291 | **0.04789** | 0.00571 | 0.11573 | **2.5e-05** | 0.15063 | 0.05301 | **0.01997** | 0.66599 | 0.52605 | 0.00884 | **0.00210** | **0.01997** | 0.09214 | 0.16966 | 0.43084 | **0.00156** | - | - |
| **Metarril** | 0.41469 | **0.03875** | **0.00081** | 0.94628 | 0.85631 | 0.38737 | 0.14471 | 0.07507 | 0.00211 | 0.20702 | **6.8e-07** | 0.33307 | 0.08139 | **0.02031** | 0.15806 | 0.96753 | 0.00596 | **2.2e-05** | **0.02409** | 0.16670 | 0.32119 | 0.87233 | **1.3e-05** | 0.52605 | - |
| **PL63** | 0.22003 | **0.00024** | 0.53966 | 0.08673 | 0.08857 | **0.01072** | 0.50995 | 0.69852 | 0.65376 | 0.32929 | 0.07508 | 0.32929 | 0.72256 | 0.96753 | **0.00179** | 0.06131 | 0.80824 | **6.5e-09** | 0.93017 | 0.41469 | 0.21843 | 0.07507 | **3.3e-09** | **0.04163** | 0.05718 |

**Table S2.** *P*-values adjusted with Bonferroni-Holmes were obtained from pairwise comparisons of survival curves of *G. plantesis* adults exposed to different concentrations of *B. bassiana* IBCB-240 and *M. anisopliae* IBCB-364 using log-rank test at *P* < 0.05 (significant *P*-values are highlighted in bold).

|  | **B_bassiana_1E+07** | **B_bassiana_1E+08** | **B_bassiana_5E+06** | **B_bassiana_5E+07** | **B_bassiana_5E+08** | **M_anisopliae_1E+07** | **M_anisopliae_1E+08** | **M_anisopliae_5E+06** | **M_anisopliae_5E+07** | **M_anisopliae_5E+08** |
| --- | --- | --- | --- | --- | --- | --- | --- | --- | --- | --- |
| **B_bassiana_1E+08** | 0.07757 | - | - | - | - | - | - | - | - | - |
| **B_bassiana_5E+06** | 0.39935 | **0.00758** | - | - | - | - | - | - | - | - |
| **B_bassiana_5E+07** | 0.37971 | 0.41994 | 0.07806 | - | - | - | - | - | - | - |
| **B_bassiana_5E+08** | **0.00020** | **0.02940** | **8.7e-06** | **0.00759** | - | - | - | - | - | - |
| **M_anisopliae_1E+07** | **0.03254** | **6.5e-05** | 0.19333 | **0.00213** | **1.3e-08** | - | - | - | - | - |
| **M_anisopliae_1E+08** | 0.65795 | 0.18272 | 0.24223 | 0.60316 | **0.00261** | **0.01363** | - | - | - | - |
| **M_anisopliae_5E+06** | **0.00125** | **4.3e-07** | **0.01615** | **4.7e-05** | **3.2e-11** | 0.30045 | **0.00049** | - | - | - |
| **M_anisopliae_5E+07** | 0.24738 | 0.59369 | **0.04171** | 0.76138 | **0.01542** | **0.00109** | 0.45688 | **1.8e-05** | - | - |
| **M_anisopliae_5E+08** | **1.1e-05** | **0.00382** | **3.6e-07** | **0.00077** | 0.38516 | **2.8e-10** | **0.00023** | **7.8e-13** | **0.00200** | - |
| **Control** | **0.00012** | **9.9e-09** | **0.00261** | **3.4e-06** | **7.8e-13** | 0.07981 | **4.8e-05** | 0.50178 | **1.0e-06** | **5.5e-14** |

**
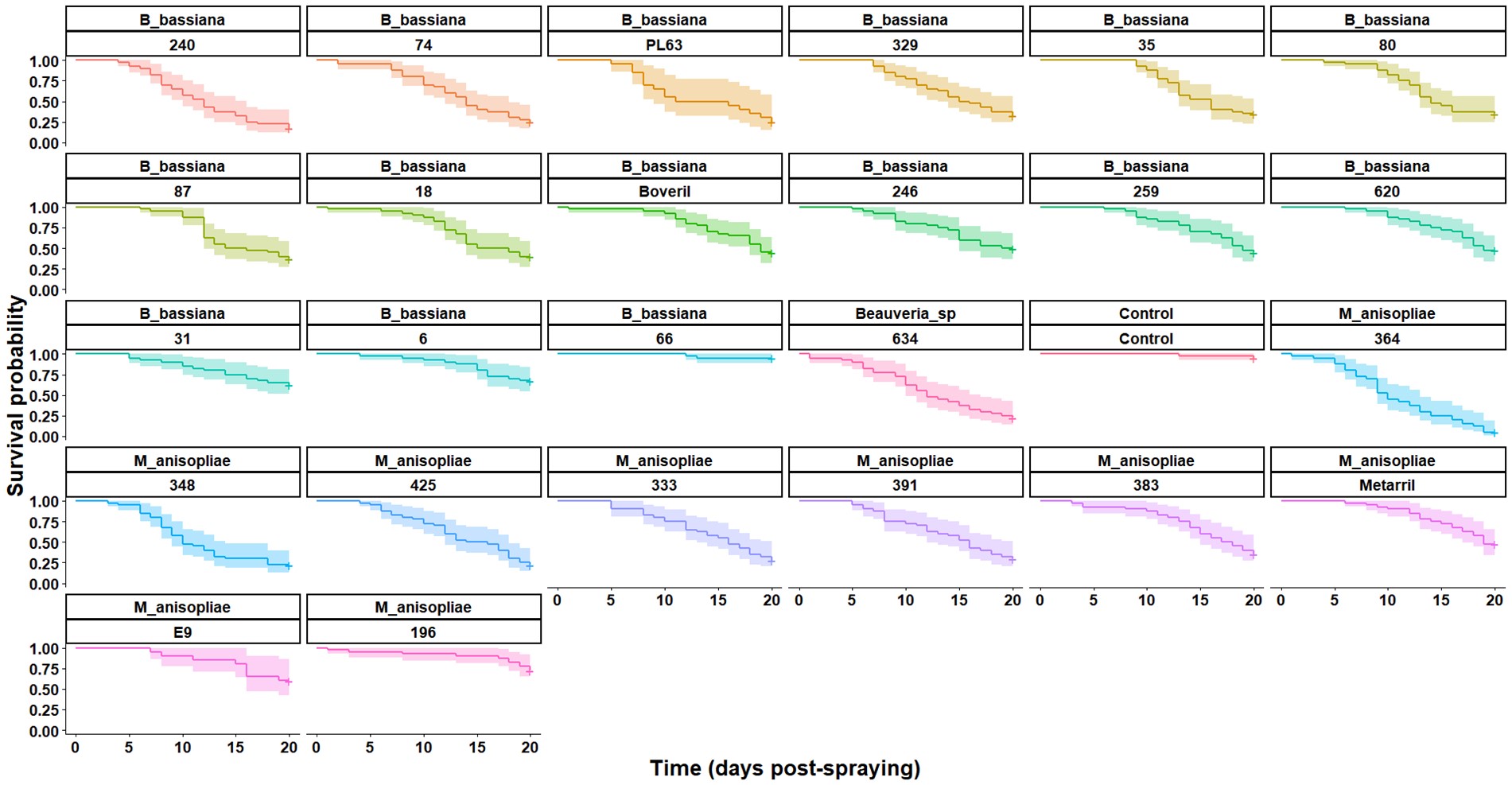
**

**Figure S1.** Kaplan-Meier estimated survival curves with their respective 95% confidence interval bands of *G. platensis* adults of several strains across *M.* *anisopliae* and *B.* *bassiana* sprayed at 1 × 10^8^ conidia mL^-1^. See Supplemental Table S1 for *P*-values of multiple pairwise comparisons between survival curves performed with log-rank test at *P* < 0.05.


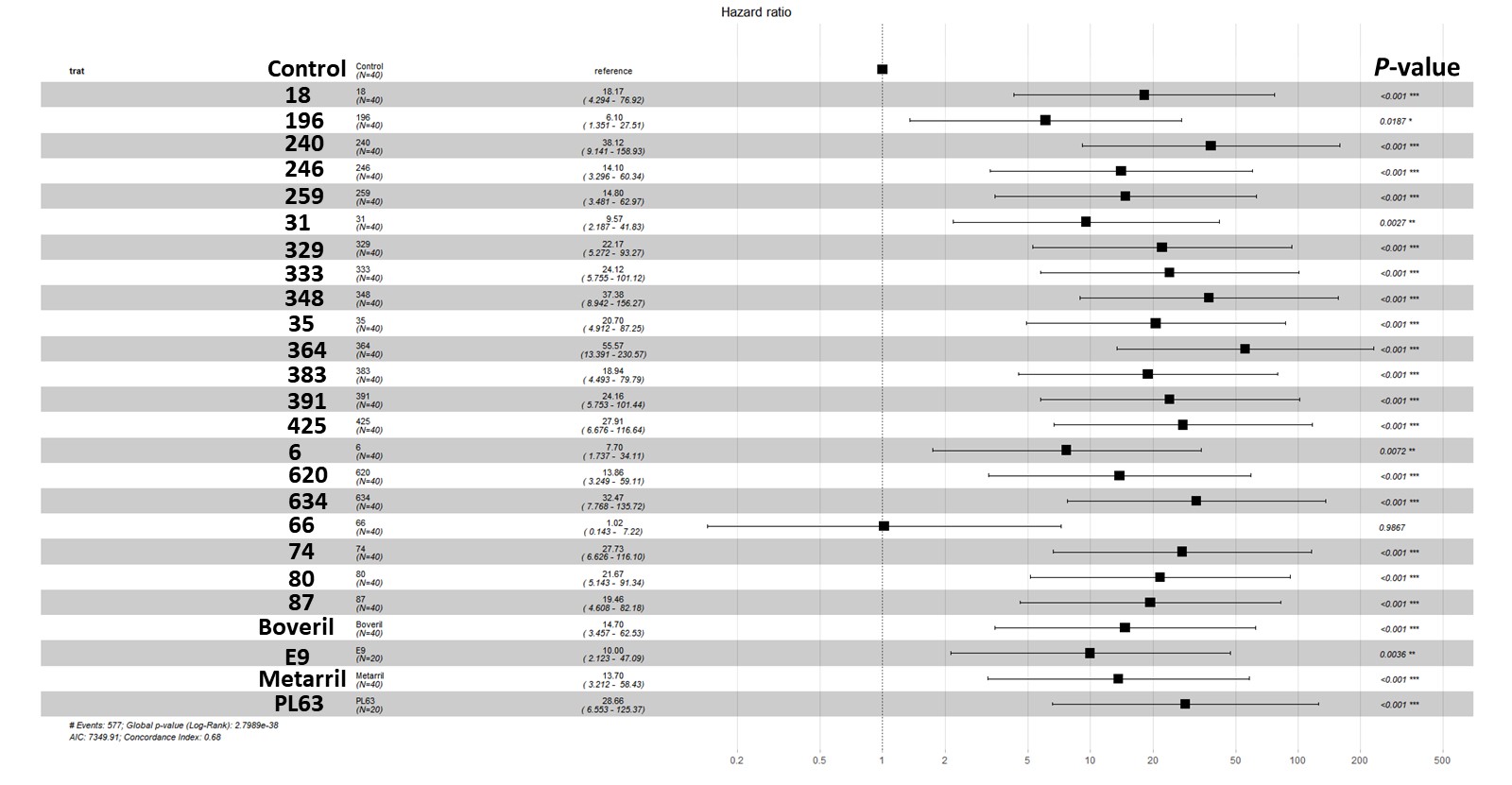


**Figure S2.** Hazard ratios (square symbols) of *G. plantesis* adults exposed to all fungal strains tested in this study, compared to the reference group assigned to be untreated weevils (control). Significant *P*-values are indicated by asterisks (*<0.05, **<0.01 or ***<0.001) and represent the strains that were significantly different (more/less virulent) from the reference group (control = untreated insects). Whiskers represent the 95% CIs.


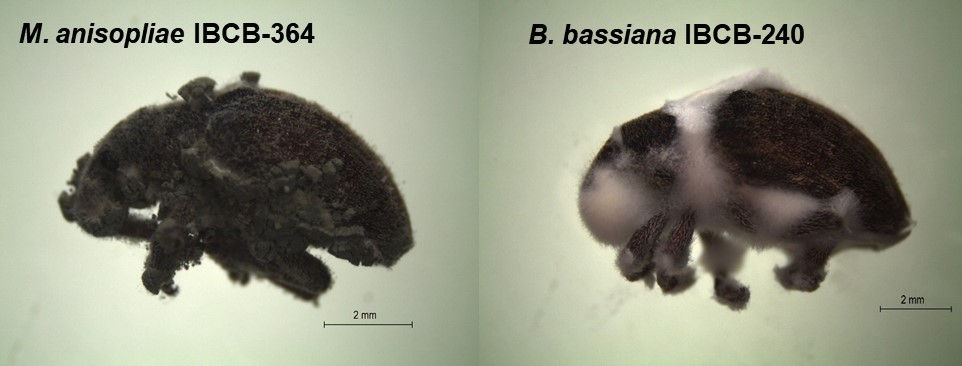


## **Figure S3.** Mycosed *G. platensis* weevils showing profuse fungal outgrowth sustaining conidiation mainly from intersegmental parts of the body, after infection with *B. bassiana* IBCB-240 and *M. anisopliae* IBCB-364.


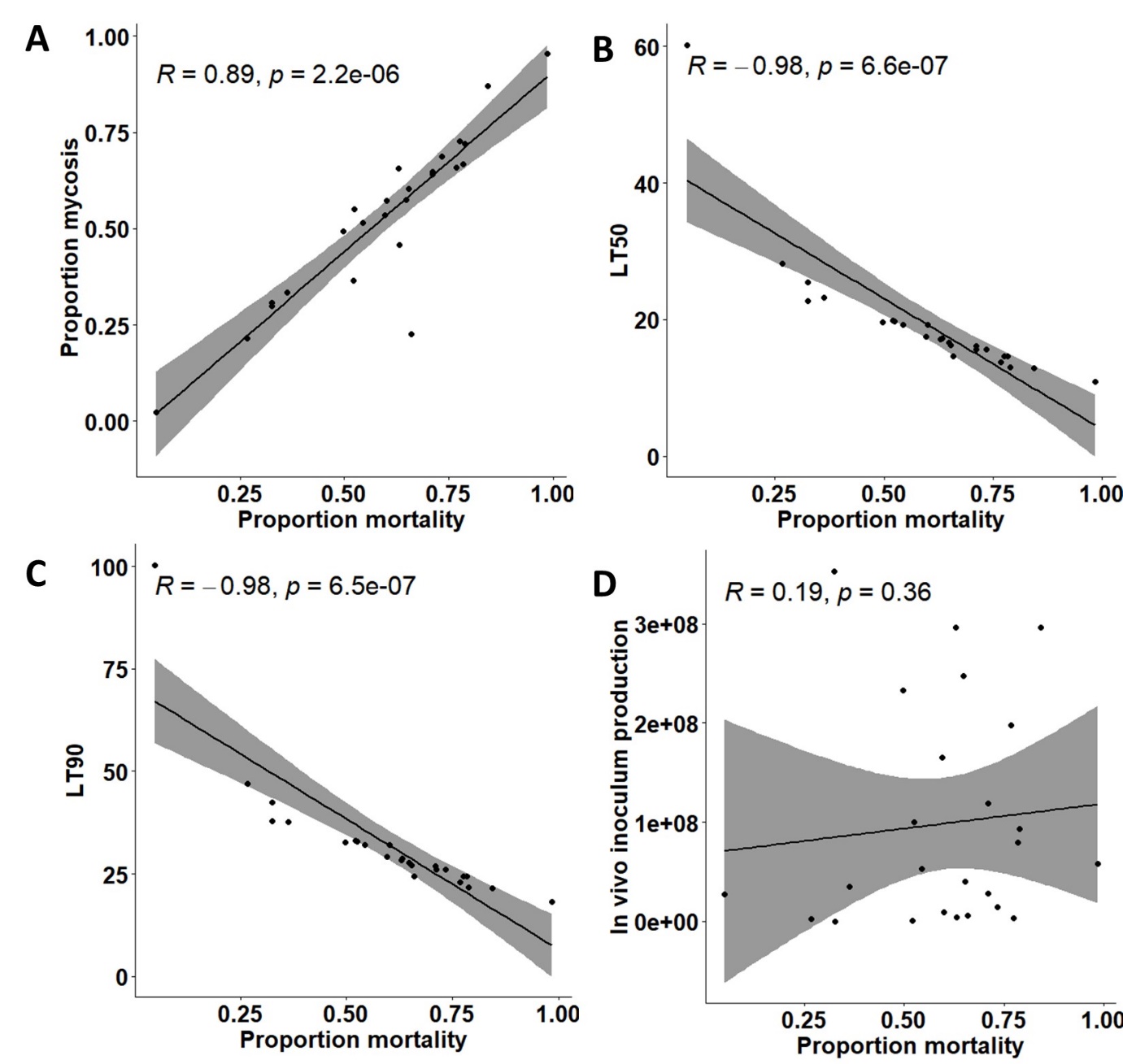


**Figure S4.** Spearman’s correlation (*r* coefficient and *P*-value) carried out to describe the relation of overall adult mortality with mycosis (confirmed mortality) (A), LT_50_ (B), LT_90_ (C) and *in vivo* inoculum production (conidia produced by weevil’s cadaver) (D). Significant correlation coefficient was attribute to *P* < 0.05, while 95% confidence bands are represented by the grey shade and black circles refer to observational points.


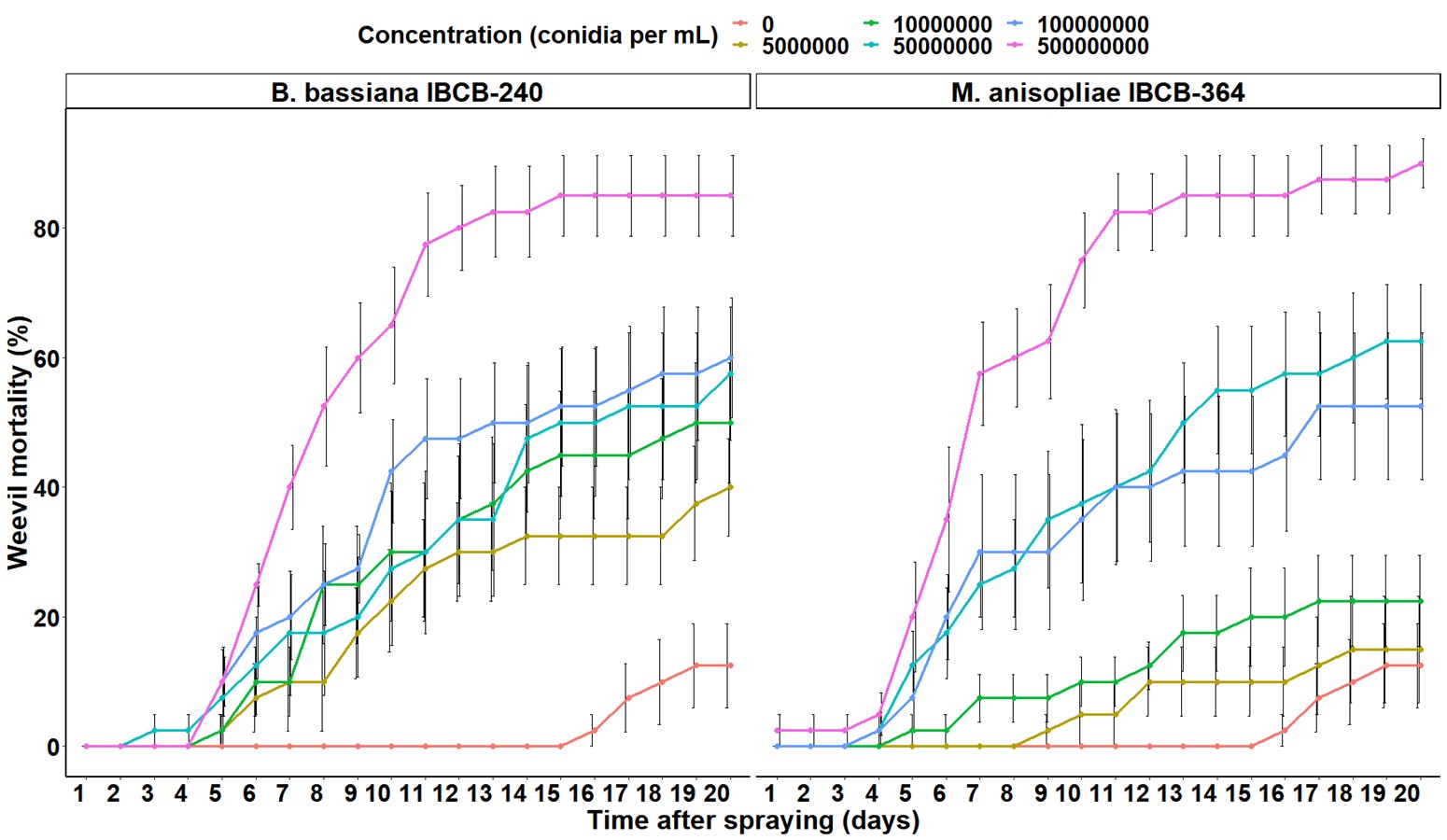


**Figure S5.** Cumulative daily proportional mortality of adult *G. platensis* weevils exposed to six spore concentrations of two fungal strains, *B. bassiana* IBCB-240 and *M. anisopliae* IBCB-364. Control (red line, concentration “0”) were exposed to only Tween 80 at 0.1%. Solid lines with different colors represent mean cumulative daily mortality rates accompanied by standard errors for each concentration with 8 replicates each and a total of 40 insects.
